# Polyurethane/Nanosilver-Doped Halloysite Nanocomposites: Thermal, Mechanical Properties, and Antibacterial Properties

**DOI:** 10.3390/polym12112729

**Published:** 2020-11-17

**Authors:** Jui-Ting Sun, Jia-Wun Li, Chi-Hui Tsou, Jen-Chieh Pang, Ren-Jei Chung, Chih-Wei Chiu

**Affiliations:** 1 Department of Materials Science and Engineering, National Taiwan University of Science and Technology, Taipei 10607, Taiwan; tommandy5566@gmail.com (J.-T.S.); a12352335@yahoo.com.tw (J.-W.L.); 2 Material Corrosion and Protection Key Laboratory of Sichuan Province, College of Materials Science and Engineering, Sichuan University of Science and Engineering, Zigong 643000, China; mayko0301@hotmail.com; 3 Department of Biotechnology, Van-Nung University, Tao-Yuan 32061, Taiwan; jcpang@mail.vnu.edu.tw; 4 Department of Chemical Engineering and Biotechnology, National Taipei University of Technology, Taipei 10608, Taiwan; rjchung@ntut.edu.tw

**Keywords:** polyurethane, halloysite, silver nanoparticles, nanocomposites, antibacterial property

## Abstract

In this study, the researchers successfully embellished the surface of halloysite (Ag/HNTs) with silver using halloysite, silver nitrate (AgNO_3_), and polyvinylpyrrolidone (PVP). The researchers then prepared polyurethane that contained pyridine ring by using 4,4′-diphenylmethane diisocyanate (MDI) and polytetramethylene glycol (PTMG) as the hard chain segment and the soft chain segment of polyurethane (PU), as well as 2,6-pyridinedimethanol (2,6-PDM) as the chain extension agent. This was followed by the preparation of Ag/HNTs/PUs nanocomposite thin films, achieved by mixing Ag/HNTs with different ratios into polyurethane that contains pyridine ring. First, the Ag/HNTs powders were analyzed using energy-dispersive X-ray spectroscopy, X-ray diffraction, and transmission electron microscopy. Subsequently, Fourier-transform infrared spectroscopy was used to examine the dispersibility of Ag/HNTs in PU, whereas the thermal stability and the viscoelasticity of Ag/HNTs/PU were examined using thermal gravimetric analysis, differential scanning calorimetry, and dynamic mechanical analysis. When the mechanical properties of Ag/HNTs/PU were tested using a universal strength tester, the results indicated a maximum increase of 109.5% in tensile strength. The researchers then examined the surface roughness and the hydrophobic ability of the Ag/HNTs/PU thin films by using atomic force microscopy and water contact angle. Lastly, antibacterial testing on *Escherichia coli* revealed that when the additive of Ag/HNTs reached 2.0 wt%, 99.3% of the *E. coli* were eliminated. These results indicated that the addition of Ag/HNTs into PU could enhance the thermal stability, mechanical properties, and antibacterial properties of PU, implying the potential of Ag/HNTs-02 as biomedicine material.

## 1. Introduction

Polyurethane (PU) is an extensively used material. It has a unique form and excellent chemical and mechanical properties [1,2]. It is widely used as insulation material for walls, rooftops, and home electrical appliances; flexible and rigid foams for furniture; thermoplastic PU for medical devices; and adhesives or sealants for the coating of clothes and shoes [3,4,5]. PU is a type of linear block copolymer composed of hard chain segment and soft chain segment. The hard chain segment is usually made up of carbamate groups [6], whereas the soft chain segment usually comprises polyether diols [7]. Different strength and hardness can be generated through changing the ratio of hard chain segment and soft chain segment or their structures or adding different chain extenders to exhibit the comprehensive properties [8]. For example, Chiu et al. [9] introduced the chain extender of 2,6-PDM into the polyurethane to prove the existence of hydrogen bond between the pyridine ring and the polyurethane, so that the shape stability of polyurethane is improved by this hydrogen bond. Numerous researchers have also mixed nanofillers into PU using the compounding method to enhance the physical properties of PU or endow PU with special properties. For example, Cai et al. [10] introduced graphene sheets into PU to endow the resultant PU with a higher level of thermal conductivity, thermal stability, and fire retardance for use in fire-retardant materials. Because of characteristics such as large body surface area, excellent physical and chemical properties, and low cost [11], clay nanotube is often used as a filler for enhancing the physical properties of polymers. Halloysite is a type of natural nanomaterial that possesses a unique nano hollow tubular structure. The ideal chemical formula of halloysite, Al_2_Si_2_O_5_(OH)_4_·2H_2_O, is composed of an external surface group of Si-O-Si and an internal surface group, Al-OH [12]. The HNTs produced in different regions have different polydispersity. In general, the length of HNTs is about 1000 nm and the diameter is between 10-70 nm [13]. Halloysite is a material that possesses excellent thermal and mechanical properties and biocompatibility. Furthermore, it is available in large amounts at low cost [14], Sikora [15] et al. added the Halloysite nanotubes into the low-density polyethylene (LDPE) to change its thermal property and mechanics property. From the results of the study, it is found that the thermal stability and maximum tensile strength can be increased through adding the Halloysite nanotubes. Since halloysite has been researched, it has been used extensively in the fields of biology and applied medicine. For example, halloysite is embedded into the base layer of bandage as a drug delivery system. Embedded halloysite can also facilitate healing in the context of burn and wound care [16] or can be injected directly to regulate drug release after it is functionalized [17]. In a recent report, Cavallaro et al. [18] made a series of comparisons of HNTs in different specific geology, and the results showed that it was demonstrated that the sizes polydispersity strongly affects the influence of HNTs as filler of nanocomposites. Additionally, many researchers have coated silver particles on inorganic substances by using physical or chemical methods, with the hope of endowing the composite materials with better physical or antibacterial properties. For example, Wu et al. [19] introduced graphene sheets into PU. In addition to endowing the composite material with better thermal and mechanical properties, the introduction of the sheets also endowed the resultant composite material with antibacterial properties.

Nanosilver is a type of antibacterial metal that possesses excellent disinfection and antibacterial properties [20] and is already used extensively in commercial products [21]. In the 1960s, silver(I) sulfadiazine was introduced, and it soon became the most well-known and extensively used silver agent applied in human medicine. It is an excellent antibacterial and is often used in treating severe burns [22,23]. Materials containing silver can also be used in eliminating microorganisms on textile fabrics [24,25], and nanoparticles also exhibit effective sterilizing rates and preservation of cell activity [26]. This indicates nanosilver offers favorable sterilizing properties. For example, Tsou et al. [27] synthesized the multiple wall carbon nanotube (MWCNT–Ag) doped with nanosilver first, and then mixed it into the polylactic acid (PLA) by melt blending way. It was found that not only the tensile strength and the thermal stability could be increased, but also the antibacterial performance could be gained. Previously, the author has reported [28] that the functional groups on the surface of carbon black are used to dope nano-silver particles on the surface of carbon black, and mixed into the polysulfone substrate. The results show that inorganic nano-particles can improve the thermal stability of the polymer. In addition, it can also provide the nano composite film with good antibacterial effect. Additionally, a large amount of biocompatible hydroxyl groups exists on the surface of halloysite [29]. These hydroxyl groups can form the hydrogen bonds with C=O in the polyurethane. It means that the HNT may have good dispersion property in the polyurethane. These hydroxyl groups can also form the ionic bonds with silver ions [30], so that the nanosilver particles can be coated on the surface of Halloysite much more evenly.

The researchers of current study prepared PU by using 4,4′-diphenylmethane diisocyanate (MDI), polytetramethylene glycol (PTMG), and 2,6-pyridinedimethanol (2,6-PDM) as the hard chain segment, the soft chain segment and the chain extension agent of PU, respectively. This was followed by the preparation of Ag/HNTs/PUs nanocomposite thin films, achieved by compounding silver-coated halloysite (Ag/HNTs) with PU. Subsequently, Fourier-transform infrared spectroscopy (FT-IR), transmission electron microscopy (TEM), and X-ray diffraction (XRD) were used to perform shape analyses of the Ag/HNTs and to examine the dispersibility of Ag/HNTs in PU. The thermal stability and viscoelasticity of the Ag/HNTs/PUs were examined using thermal gravimetric analysis (TGA), differential scanning calorimetry (DSC), and dynamic mechanical analysis (DMA). The mechanical properties of the Ag/HNTs/PU were then tested using a universal strength tester, followed by examination of surface roughness and the hydrophilic and hydrophobic abilities of the Ag/HNTs/PU thin films by using atomic force microscopy (AFM) and water contact angle (WCA). Lastly, the antibacterial properties of the Ag/HNTs/PUs were tested using *Escherichia coli (E. coli)*.

## 2. Experimental

### 2.1. Materials

4,4′-Diphenylmethane diisocyanate (MDI), 2,6-pyridinedimethanol (2,6-PDM), and halloysite nanotubes were purchased from Sigma-Aldrich (St. Louis, MO, USA). Polyvinylpyrrolidone and silver nitrate were purchased from Echo Chemical (Echo Chemical CO., LTD., Miaoli County, Taiwan). PTMG (Mw = 1000) was obtained from Sigma-Aldrich. *N,N*-dimethylacetamide (DMAc) was obtained from Mallinckrodt Chemicals (Dublin, Ireland). All the raw materials used were synthetic grade.

### 2.2. Preparation of Ag/HNTs Powder

First, 1 g of halloysite powders and 100 mL of dimethylformamide were mixed and placed in an ultrasonic vibration machine to vibrate for 10 min. Next, polyvinylpyrrolidone was added as dispersing agent and the mixture was vibrated for another 30 min. After the completion of the vibration process, a mixture solution consisting of 10 g of silver nitrate (AgNO_3_) and deionized water was added, and the mixture was stirred at 80 °C for 12 h. The preparation process of nano-Ag/PVP/HNTs was thus completed. Lastly, the mixture solution was centrifuged at 12,000 rpm. The lower level precipitates were taken out to extract the nano-Ag/HNTs, and the extracted nano-Ag/HNTs samples were rinsed three times to obtain pure nano-Ag/HNTs powders.

### 2.3. Synthesis of Ag/HNTs/PU Nanocomposites

**Scheme 1:**
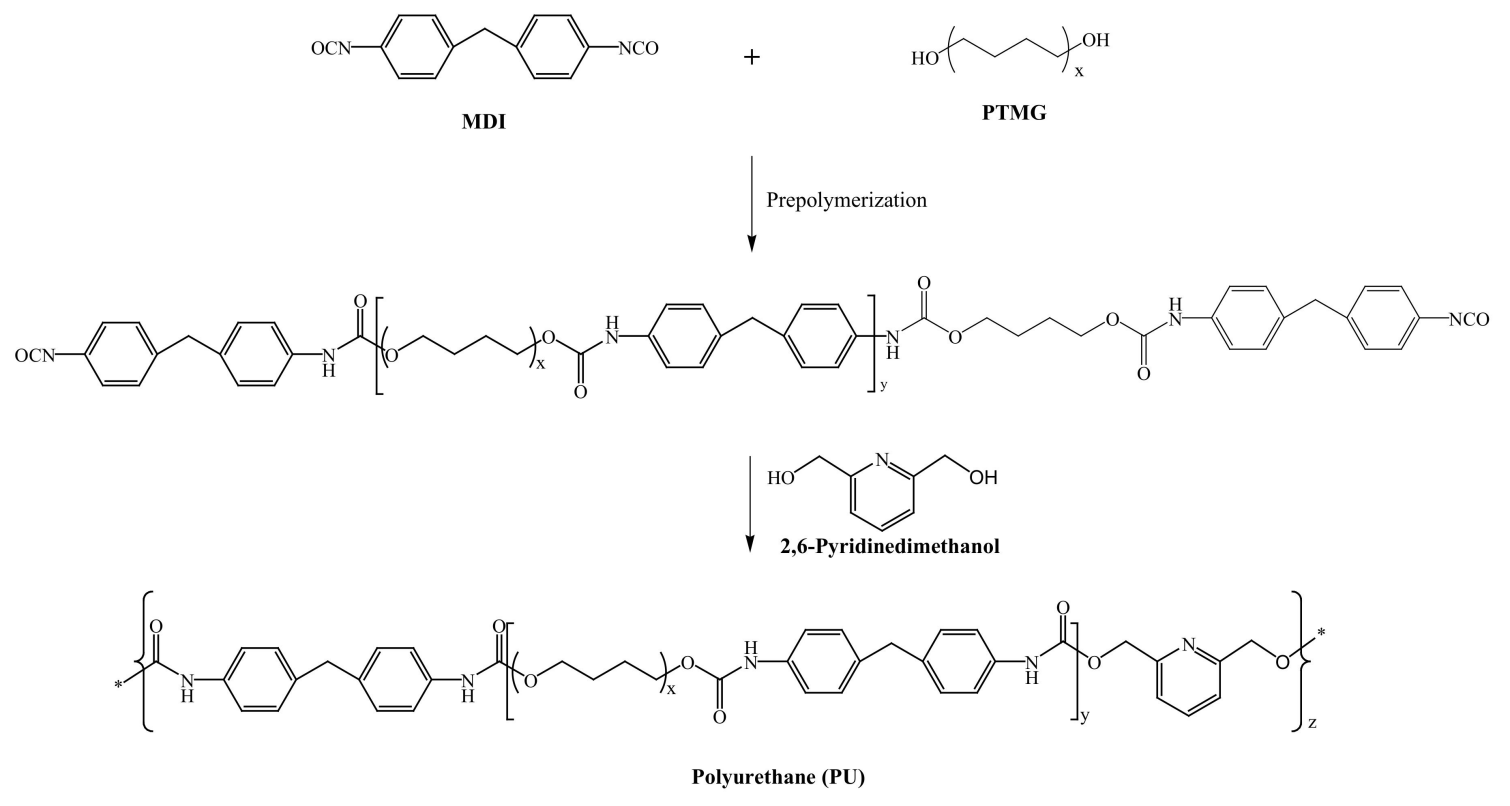
Synthesized image of polyurethane (PU).

The Ag/HNTs/PU nanocomposites were synthesized using a two-step method. First, MDI, PTMG, and DMAc were added to a 500 mL three-necked, flat-bottomed flask fitted with a nitrogen inlet. The reaction proceeded by heating the mixture to 80 °C and stirring at 200 rpm. PU prepolymer formed after reacting for 2 h. Second, 2,6-PDM in DMAc was added to the flask, and the reaction proceeded for 1 h. The mixture was then added to Ag/HNTs powder that had been subjected to 1 h of ultrasonic vibration, and the reaction proceeded for 1 h. The mixture was then collected and defoamed using a vacuum pump, and Ag/HNTs/PU films were obtained by pouring a suitable amount of the Ag/HNTs/PU solution onto Teflon plates. The liquid films were dried in a temperature-programmable circulating oven for 8 h. The synthesizing process is illustrated in Scheme 1. The experimental formula is as compiled and presented in Table 1.

### 2.4. Fourier Transform Infrared Spectroscopy (FT-IR)

FT-IR was performed using a PerkinElmer spectrometer (Model Spectrum One, MA, USA) with a scanning range of 4000–650 cm^−1^ and a resolution of 2 cm^−1^. The values of 16 scans were averaged.

### 2.5. Energy-Dispersive X-ray Spectroscopy Observations (EDS)

Element compositions of the Attapulgite/Chitosan membrane were examined by energy dispersive X-ray spectroscopy (EDS) (model 7021-H; Horiba, Tokyo, Japan). Specimens of 2 × 2 cm^2^ were fixed on a sample holder using conductive adhesive tape and then coated with a thin layer of gold to improve image resolution. The samples were photographed at 1000 magnification.

### 2.6. Transmission Electron Microscopy (TEM)

A transmission electron microscope (TEM, Hitachi model H-7500, Tokyo, Japan) was used to examine the morphology of the Ag/HNTs/PUs nanocomposites. The samples for TEM examination were first prepared by placing the nanocomposite films into epoxy capsules and curing the epoxy at 70 °C for 24 h in an oven. The cured epoxies containing Ag/HNTs/PUs nanocomposites were then microtomed with a diamond knife into 70–90-nm-thick slices at −100 °C. Finally, a 3-nm-thick carbon layer was deposited on the slices placed on 200-mesh copper grids for TEM observation.

### 2.7. X-ray Diffraction (XRD)

X-ray diffractometer measurements were performed using a Rigaku diffractometer (BRUKER, model D2 Phaser XRD, MA, USA). X-rays were filtered through CuKα radiation tubes using Ni filters, and tests were conducted at 60 Kv and 300 mA. The results showed 0.05° per point and a scanning range of 2θ = 10°–70°.

### 2.8. Thermogravimetric Analysis (TGA)

Thermogravimetric analyses were performed using a PerkinElmer (model Pyris 1). The samples (weighing 5–8 mg) were subjected to a nitrogen environment and an initial temperature of 50 °C; the temperature was then increased in 10 °C/min increment to 700 °C, after which analyses were made. Each reported TGA is the mean of decomposition temperatures measured for three to four specimens.

### 2.9. Differential Scanning Calorimetry (DSC)

DSC was performed using a PerkinElmer calorimeter (model Jade). At first, the samples were heated up to 100 °C with a heating rate of 50 °C/min, maintained for 3min to eliminate the heat history, and then cooled down to −80 °C with a cooling rate of 10 °C/min and maintained for 5 min. Finally, the samples were heated up to 50 °C with a heating rate of 10 °C/min. Transitions were recorded from the cooling scan and the second heating scan. The glass transition temperatures (Tg) were located as the midpoints of the sharp descent regions in the recorded curves. All tested Ag/HNTs/PU samples were used in masses of 5–8 mg.

### 2.10. Dynamic Mechanical Analysis (DMA)

DMA was performed using a Seiko dynamic mechanical spectrometer (Model SII Muse, DMS6100, Tokyo, Japan). The Ag/HNTs/PU films had dimensions of 20 × 5 × 0.2 mm^3^ (L × W × H). The stress test conducted between −50 and 50 °C had the following parameters: 1-Hz frequency, 5-μm amplitude, and 3 °C/min heating rate.

### 2.11. Stress–Strain Testing

Tensile strength and elongation at break were measured using a universal testing machine (MTS QTEST5, model QC505B1). Testing was conducted according to ASTM D638 specifications. The dimension of the film specimens was 45 mm × 8 mm × 0.2 mm.

### 2.12. Contact Angle

Contact angles between the samples and deionized water were measured using a Face instrument (model CA-VP150) at room temperature. Dynamic advancing and receding contact angles were recorded while water was added to and withdrawn from the drop, respectively, using a syringe pump. Each reported contact angle is the average value from 3 to 4 drops.

### 2.13. Surface Roughness Analysis

Atomic force microscopy (AFM) scans were performed using a CSPM5500 instrument from Being Nano-instruments. Generally, there are two types of imaging modes, i.e., tapping and contact modes. Tapping mode was used in this study such that the oscillating probe cantilever causes the tip to make only intermittent contact with the sample. With respect to the phase of the sine wave driving the cantilever, the phase of the tip oscillation is highly sensitive to various sample surface characteristics. Therefore, the tip can also sense the phase images of the sample surface in addition to the topography. The specimens were cut from Ag/HNTs/PUs nanocomposite films with different clay contents.

## 3. Results and Discussion

### 3.1. Characterization of Ag/Halloysite

**Figure 1:**
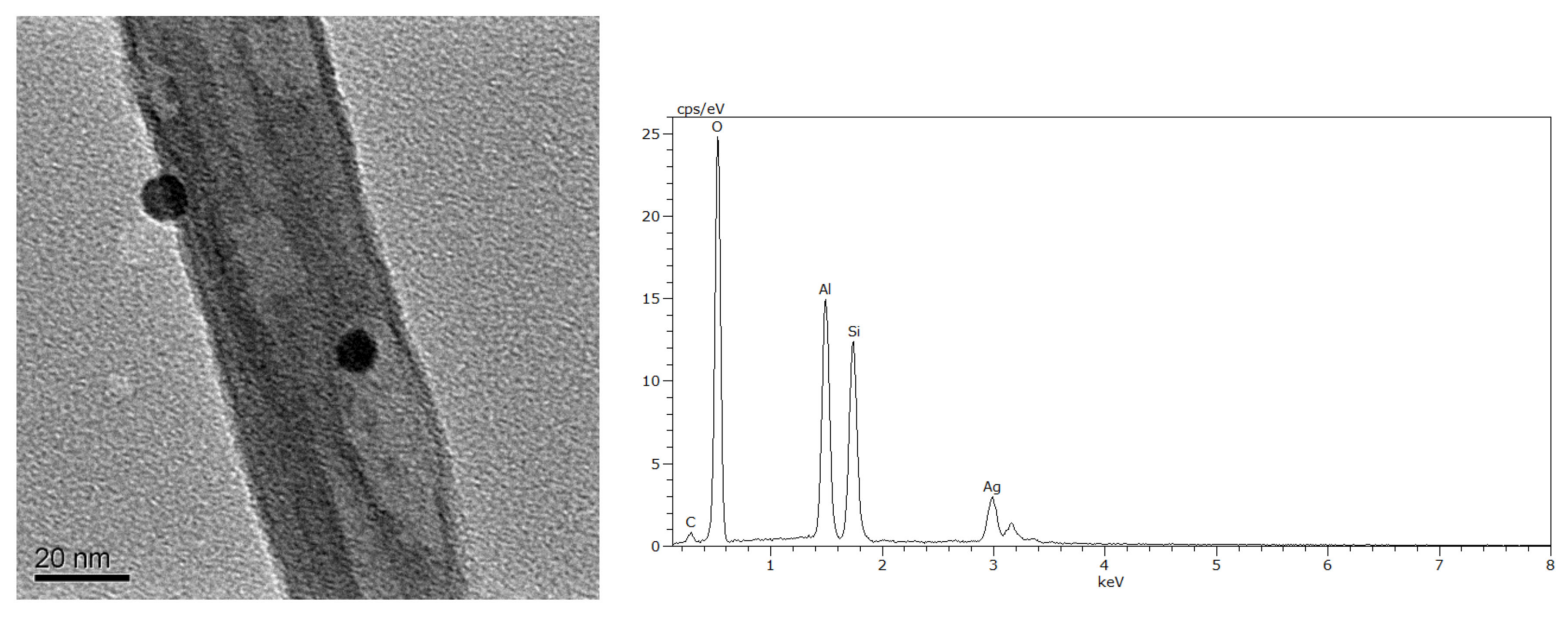
Transmission electron microscopy (TEM) and energy dispersive X-ray spectroscopy (EDS) images of the Ag/Halloysite.

**Figure 2:**
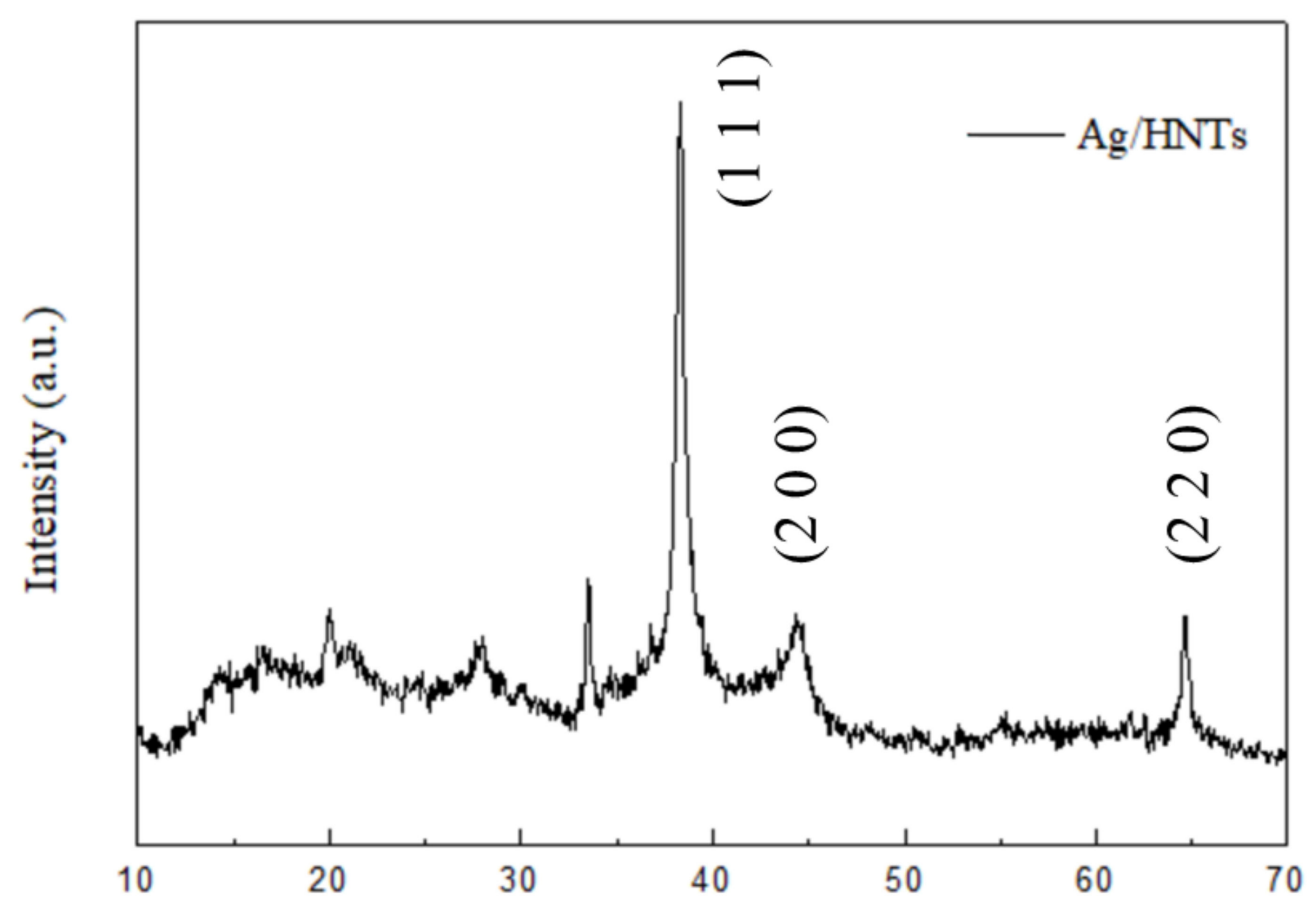
X-ray diffraction (XRD) patterns of the Ag/Halloysite.

Figure 1 presents the surface structure of the Ag/halloysite powder, captured through TEM, and the Ag element content chart measured using EDS. The bar structure of Ag/HNTs were observed through TEM, where TEM revealed that the ion-interface interactions formed between Ag^+^ in AgNO_3_ and –OH on the surface of HNTs can be used to form silver nanoparticles on the surface of HNTs by in-situ reduction method. The calculation of EDS indicated that the silver (Ag) content of the Ag/halloysite was 13.96%; XRD was used to assay the Ag/halloysite powder, and the results are displayed in Figure 2. In the 38.1° (2θ), 44.3° (2θ), and 64.4° (2θ) portions of the figure, the classic stretching vibration peak of silver can be observed. The results were similar to those obtained in relevant studies [31,32].

### 3.2. Fourier Transform Infrared Spectroscopy

**Figure 3:**
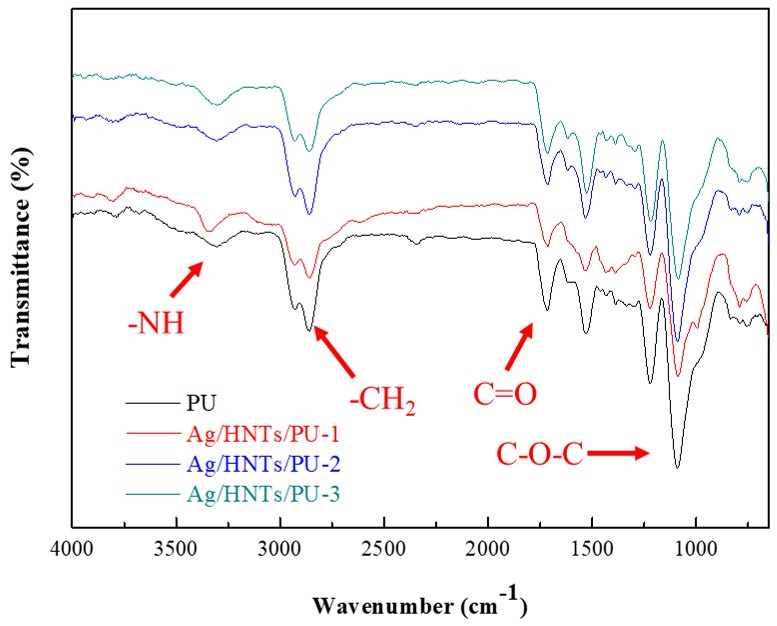
Fourier-transform infrared spectroscopy (FT-IR) spectra of the Ag/HNTs/PUs at the wavenumber range of 4000–650 cm^−1^.

Figure 3 depicts the FT-IR spectra of Ag/HNTs/PUs in a wavenumber range of 4000–650 cm^−1^. Five classic PU(–COONH) peaks were observed in the pure PU. An N–H stretching vibration peak was observed at 3356 cm^−1^; symmetrical and asymmetrical CH_2_ stretching vibration peaks were observed at 2939 and 2855 cm^−1^; a C=O stretching vibration peak appeared at 1713 cm^−1^; and a C–O–C stretching vibration peak appeared at 1107 cm^−1^. No N=C=O absorption peak was present near 2275–2240 cm^−1^; this indicated that the reaction of 2,6-PDM and MDI was almost near completion. The results also indicated that no changes occurred in the stretching vibration peak after the addition of Ag/HNTs.

### 3.3. Energy-Dispersive X-ray Spectroscopy Observations

**Figure 4:**
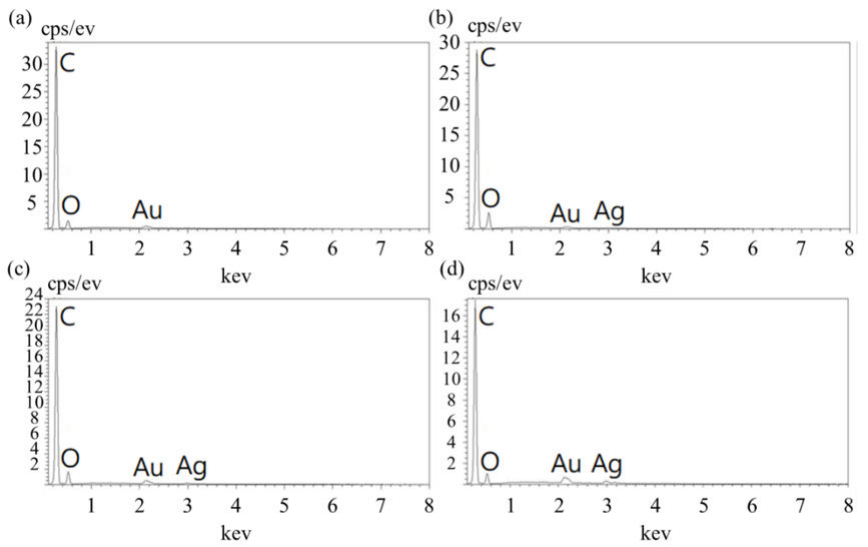
EDS images of the Ag/HNTs/PUs (**a**) PU (**b**) Ag/HNTs/PU-1 (**c**) Ag/HNTs/PU-2 (**d**) Ag/HNTs/PU-3.

After Ag/HNTs was added to the PU, the Ag content was analyzed using EDS elemental analysis. The results are presented in Figure 4; the content of the main element was calculated and compiled and is presented in Table 2. The Ag content of Ag/HNTs/PU-1, Ag/HNTs/PU-2, and Ag/HNTs/PU-3 was 1.02 wt%, 1.95 wt%, and 3.96 wt%, respectively. The Ag element in the Ag/HNTs/PU increased with Ag/HNTs content. Thus, the larger the additive amount of Ag/HNTs, the larger the amount of Ag dispersed in the PU. The analysis indicated that the nanosilver particles were adsorbed well on the HNTs and dispersed evenly in the PU thin films.

### 3.4. X-ray Diffraction

**Figure 5:**
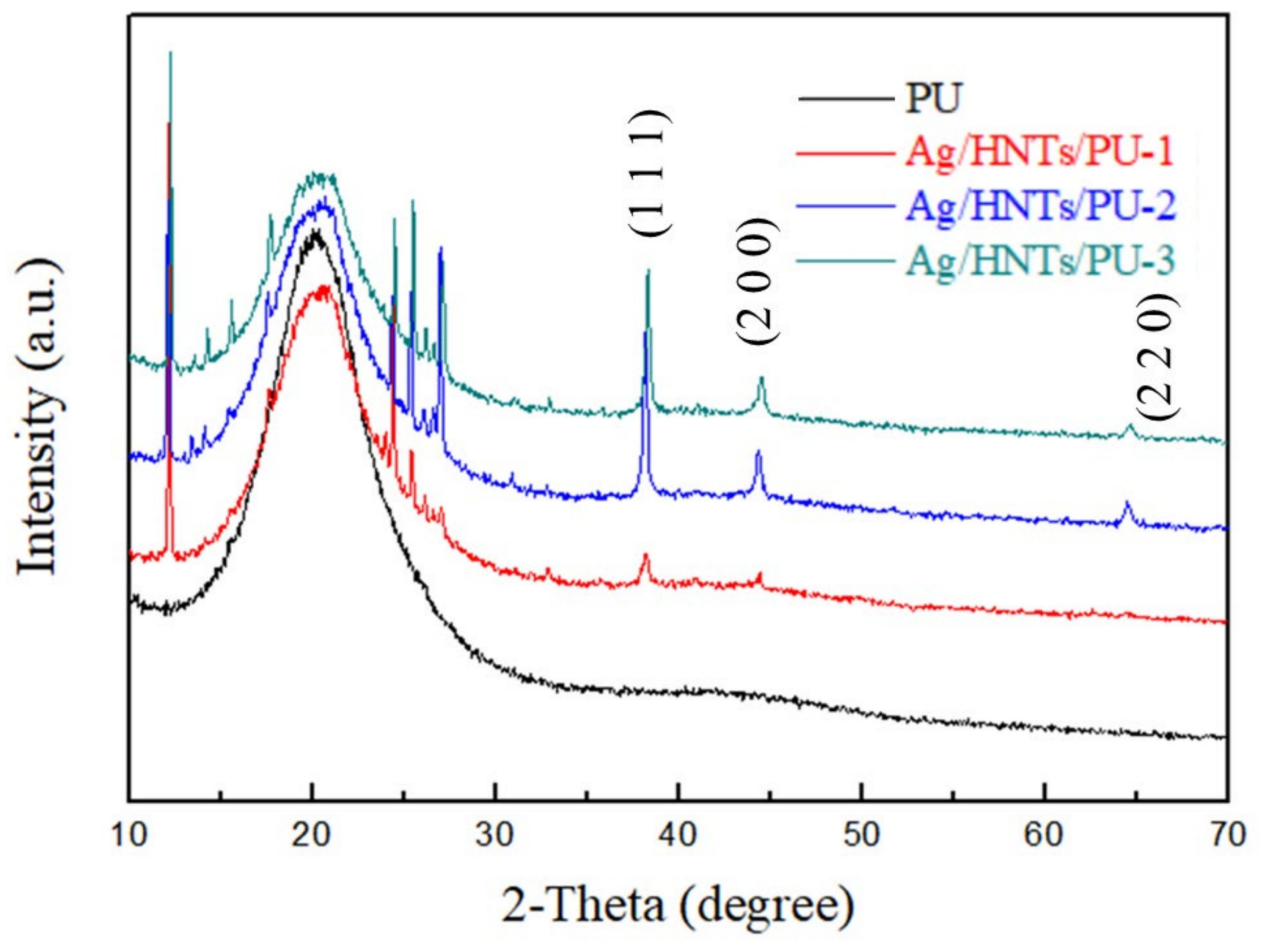
XRD patterns of the Ag/HNTs/PUs.

The XRD spectra of the Ag/HNTs/PUs composites are reproduced in Figure 5. The spectrogram indicated that the PU existed in amorphous state. After the addition of the Ag/HNTs/PU-1, Ag/HNTs/PU-2, and Ag/HNTs/PU-3, all exhibited characteristic crystallization peaks at 2θ = 12°, 25°, and 26°. The characteristic crystallization peak of silver element is 2θ = 38.1°, 44.3°, and 64.4°, respectively. These results are similar to the results obtained in the testing of the Ag/HNTs powder; the crystallization peak became more prominent in HNTs additive amount.

### 3.5. Thermal Properties of Ag/HNTs/PU Nanocomposites

**Figure 6:**
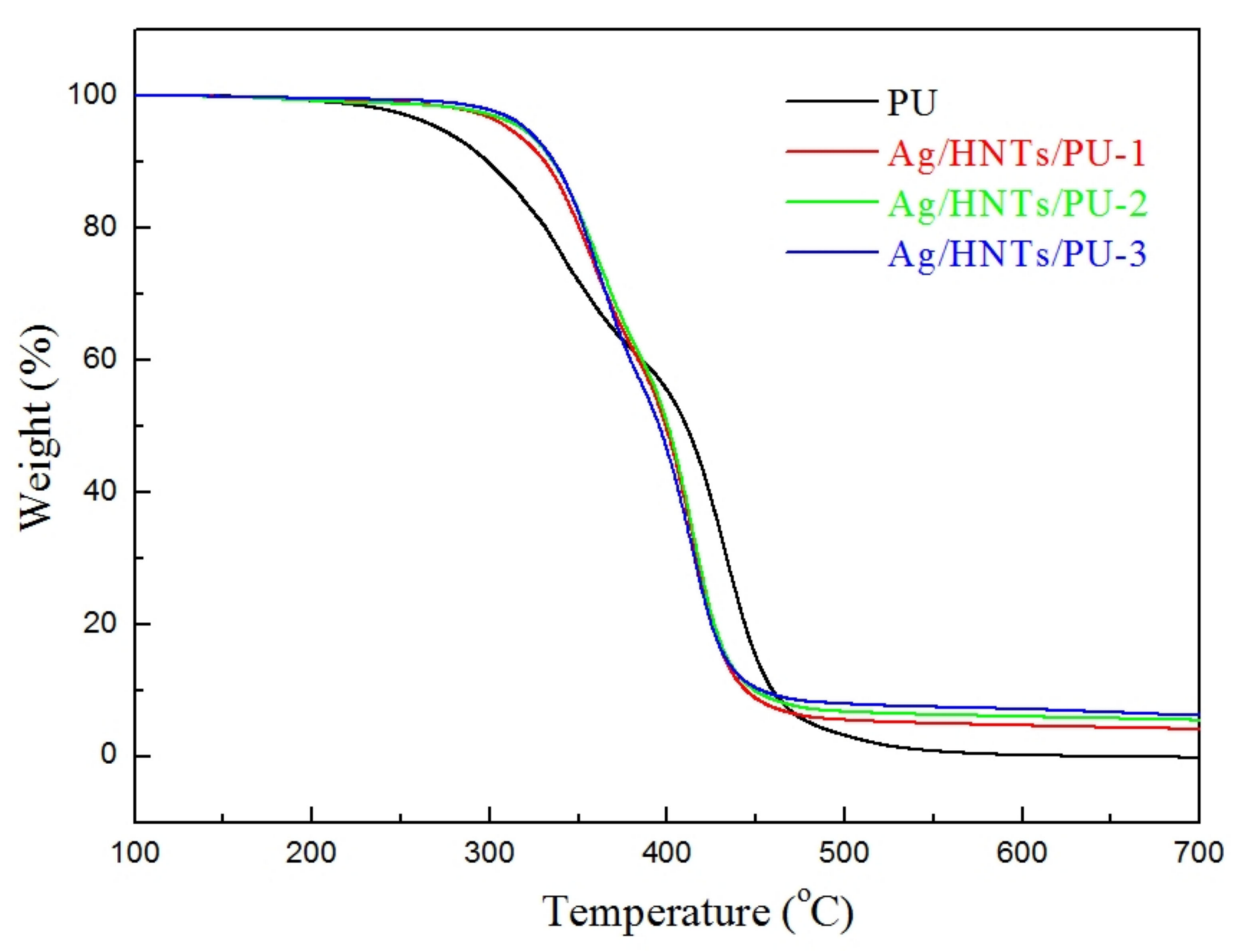
Thermal gravimetric analysis (TGA) curves of the Ag/HNTs/PUs.

The thermal analysis curve of Ag/HNTs/PUs is presented in Figure 6. The initial decomposition temperature, as well as the residual volume at 700 °C, were calculated and are presented in Table 3. The thermal decomposition temperatures of PU, Ag/HNTs/PU-1, Ag/HNTs/PU-2, and Ag/HNTs/PU-3 were 291.0 °C, 323.7 °C, 329.0 °C, and 330.1 °C, respectively. The residual volumes of PU, Ag/HNTs/PU-1, Ag/HNTs/PU-2, and Ag/HNTs/PU-3 at 700 °C were 0.2%, 4.1%, 5.4%, and 6.1%. When Ag/HNTs was added to pure PU, the thermal decomposition temperature of PU increased to a maximum temperature of 39.1 °C, and the residual volume of PU at 700 °C also increased, from 0.2% to 6.1%; this was because the inorganic fillers in the study possessed a higher level of thermal stability than did PU, and it has been reported in the literature that when the inorganic filler is uniformly dispersed in the matrix, it can form the barrier effect to the volatile products of polymer degradation [33]. Therefore, the addition of these inorganic fillers to PU could result in the enhancement of the PU’s stability. The addition of Ag/HNTs to PU thin films could effectively enhance the thermal stability of the PU.

**Figure 7:**
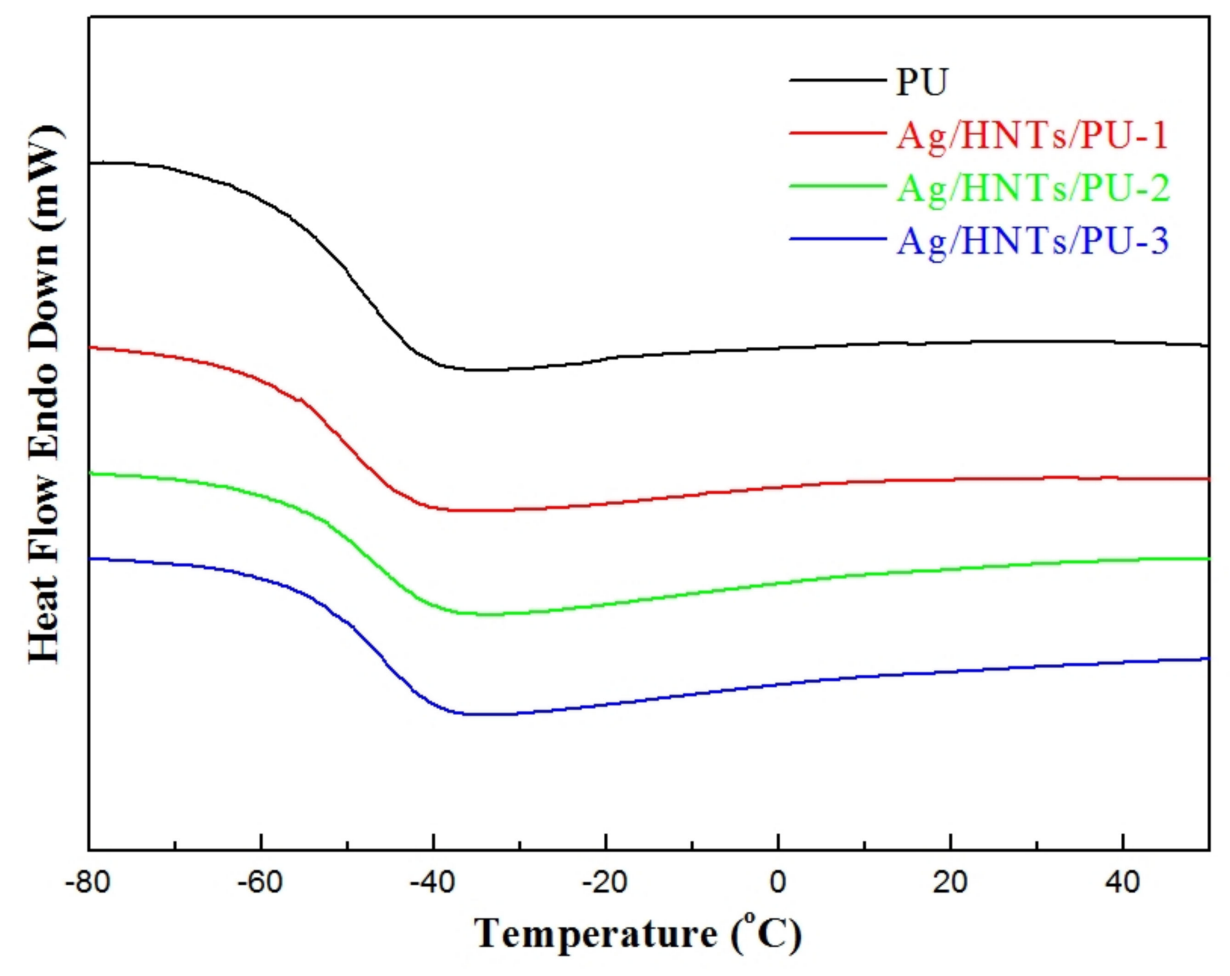
Differential scanning calorimetry (DSC) thermograms of the Ag/HNTs/PUs.

The DSC curves of the Ag/HNTs/PUs are presented in Figure 7, and the Tg points of the Ag/HNTs/PUs are presented in Table 3. The data indicated that the Tg points of Ag/HNTs/PU-1, Ag/HNTs/PU-2, and Ag/HNTs/PU-3 were −51.5 °C, −50.7 °C, −48.9 °C, and −47.4 °C, respectively. The glass transition temperature increased with the amount of Ag/HNTs added; this phenomenon was similar to that observed with DSC. It may have occurred because when the inorganic materials (i.e., Ag/HNTs) were added to the PU, the entrapment of the polymer into lumen of HNTs [34], and they effectively hindered the activity. Therefore, a high level of energy was needed to cause activity in the PU molecular chain, and this in turn resulted in the increase in glass transition temperature.

### 3.6. Dynamic Mechanical Analysis

**Figure 8:**
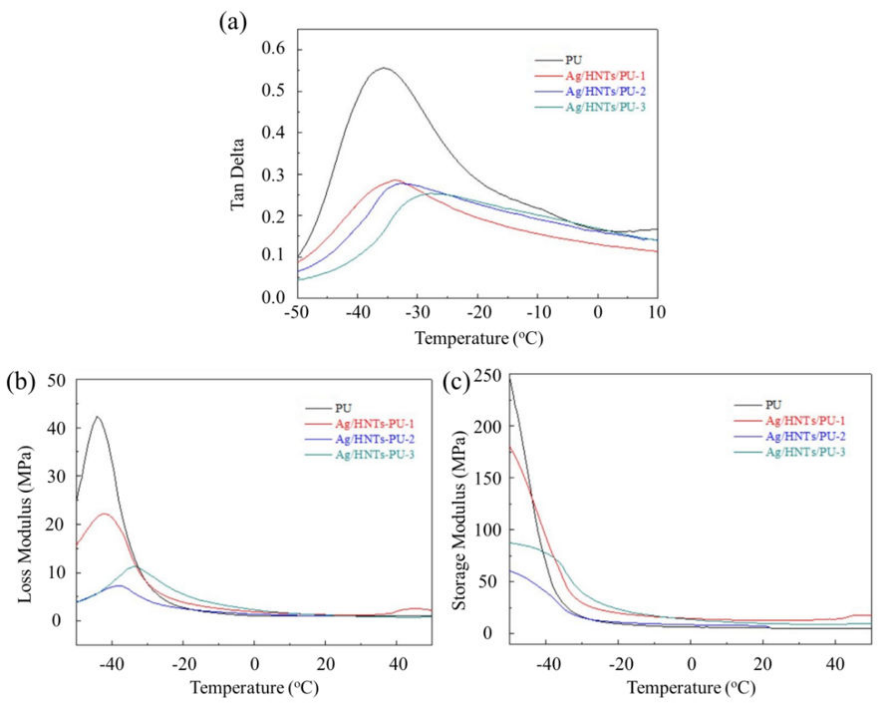
Dynamic mechanical analysis of the Ag/HNTs/PU, (**a**) Tanδ, (**b**) loss modulus (E″), (**c**) storage modulus.

The DMA curves of the Ag/HNTs/PUs are presented in Figure 8. The maximum value of T_gd_ and Tan δ were calculated using the loss modulus (E′′) and are presented in Table 4. The results indicated that the T_gd_ values of PU, Ag/HNTs/PU-1, Ag/HNTs/PU-2, and Ag/HNTs/PU-3 calculated based on E′′ were −44.09 °C, −42.27 °C, −37.68 °C, and −33.72 °C, whereas the T_gd_ values of PU, Ag/HNTs/PU-1, Ag/HNTs/PU-2, and Ag/HNTs/PU-3 calculated using Tanδ were −35.68 °C, −33.77 °C, −32.73 °C, and −27.90 °C, respectively. The addition of Ag/HNTs led to increases of the T_gd_ point; this phenomenon was similar to that observed with DSC. It may have occurred because the Ag/HNTs effectively hindered PU molecular chain activity, increasing the T_gd_ point. The Tanδ curves indicated that the maximum Tanδ of PU, Ag/HNTs/PU-1, Ag/HNTs/PU-2, and Ag/HNTs/PU-3 were 0.5568, 0.2854, 0.2772, and 0.2530, respectively. The addition of Ag/HNTs resulted in a lower level of loss modulus and a higher level of storage modulus. The results also indicated that the addition of Ag/HNTs resulted in a higher level of elasticity and a lower level of viscosity in the PU.

### 3.7. Stress–Strain Testing

**Figure 9:**
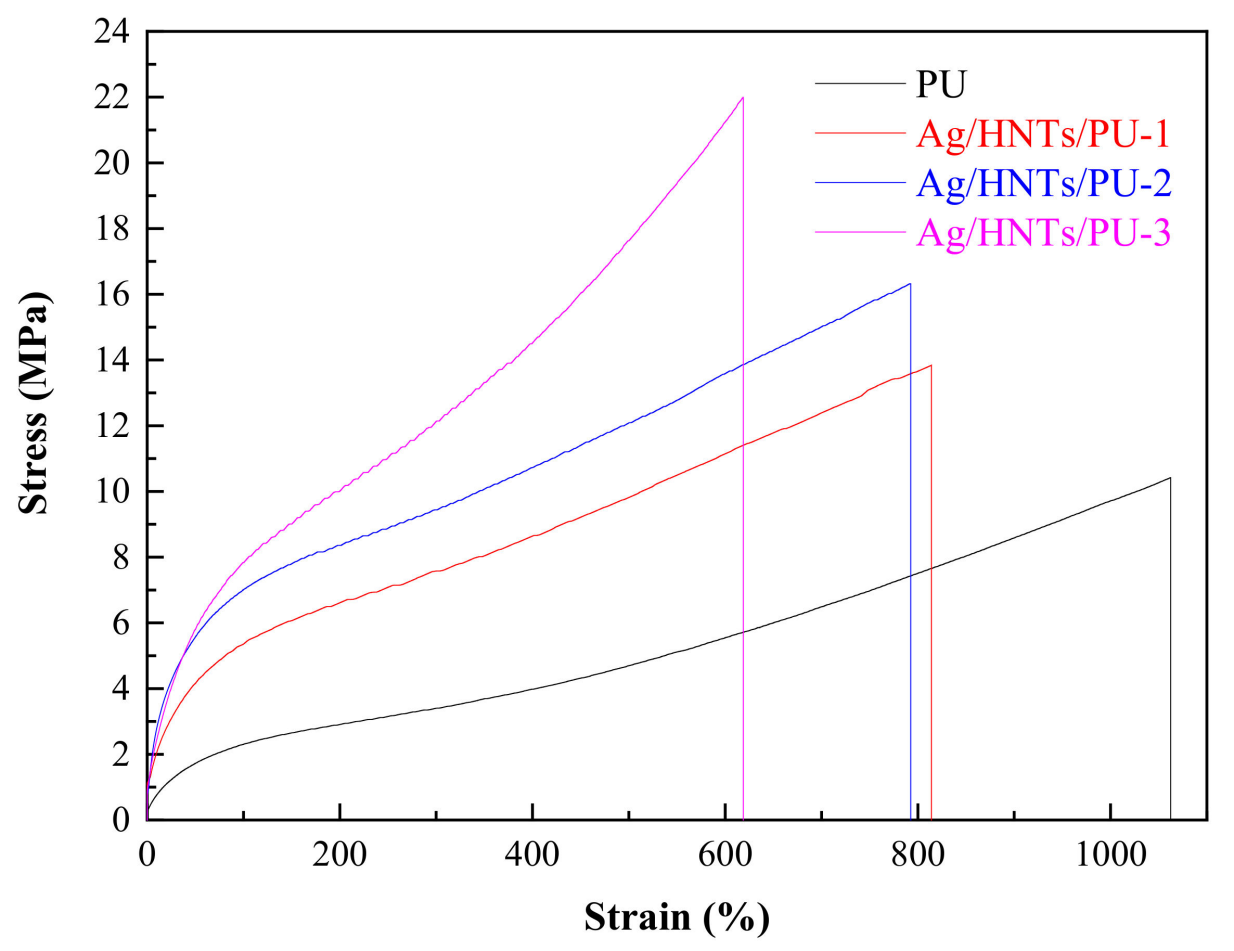
Tensile properties of the Ag/HNTs/PUs stress–strain curve.

The stress–strain curves of Ag/HNTs/PUs are provided in Figure 9. These results were used to calculate the maximum stress, the elongation at break, and the Young’s modulus, and the results are presented in Table 5. The data indicated that the maximum stress, elongation at break, and Young’s modulus of the original untreated PU were 10.5 MPa, 1060%, and 0.78 MPa, respectively. After the addition of Ag/HNTs, the maximum stress of the resulting Ag/HNTs/PUs increased from 10.5 MPa to 22.0 MPa. However, the elongation at break decreased from 1060 to 618%, and Young’s modulus increased from 0.78 MPa to 2.82 MPa. The addition of Ag/HNTs enhanced the mechanical properties of the Ag/HNTs/PUs and resulted in improved PU rigidity. The rigidity caused by the addition of inorganic materials to PU is a classic characteristic of organic/inorganic composite materials.

### 3.8. Surface Properties of Ag/HNTs/PU Nanocomposites

**Figure 10:**
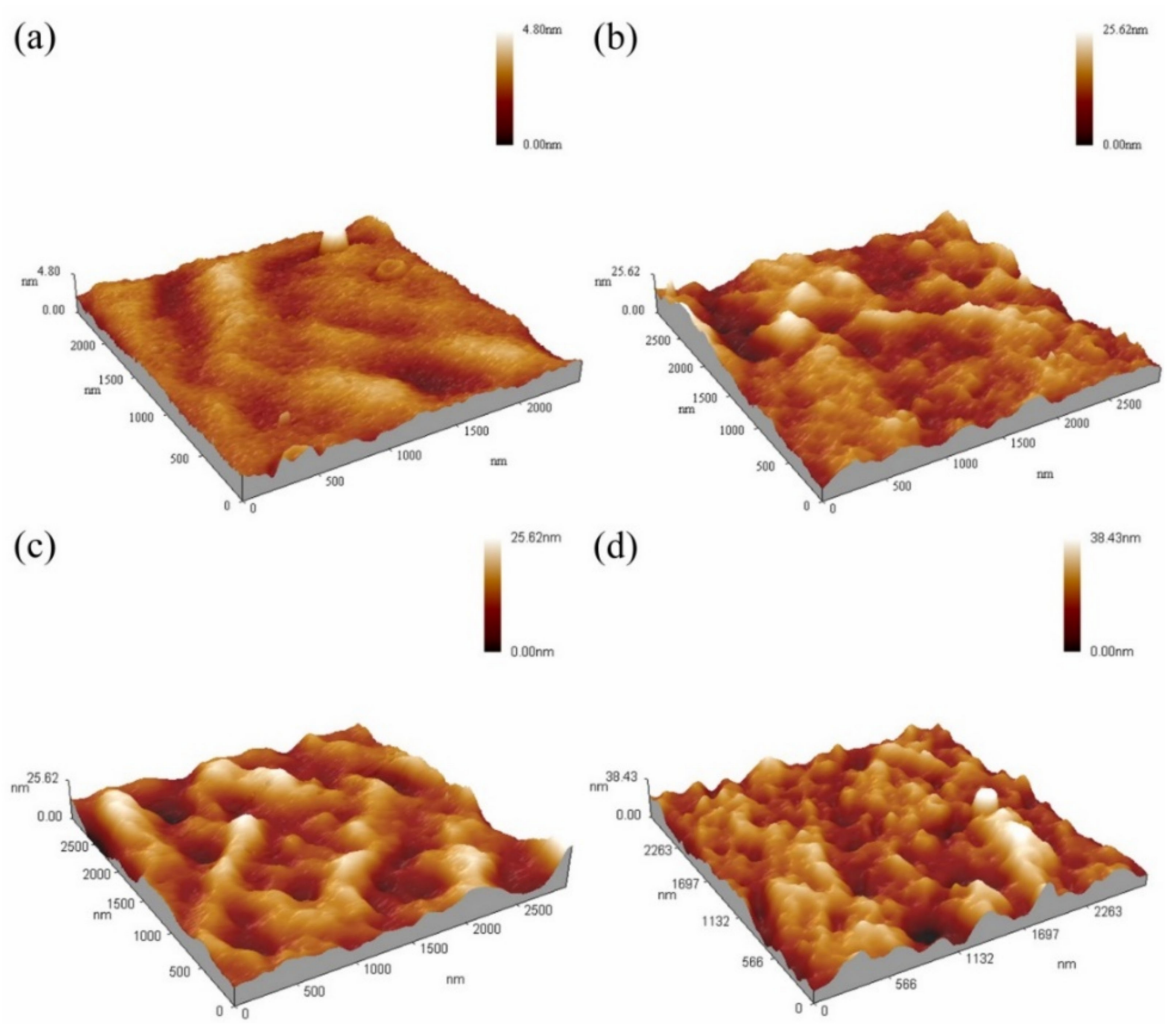
The 3D morphologies of the surfaces of Ag/HNTs/PUs nanocomposites (**a**) PU (**b**) Ag/HNTs/PU-1 (**c**) Ag/HNTs/PU-2 (**d**) Ag/HNTs/PU-3.

**Figure 11:**
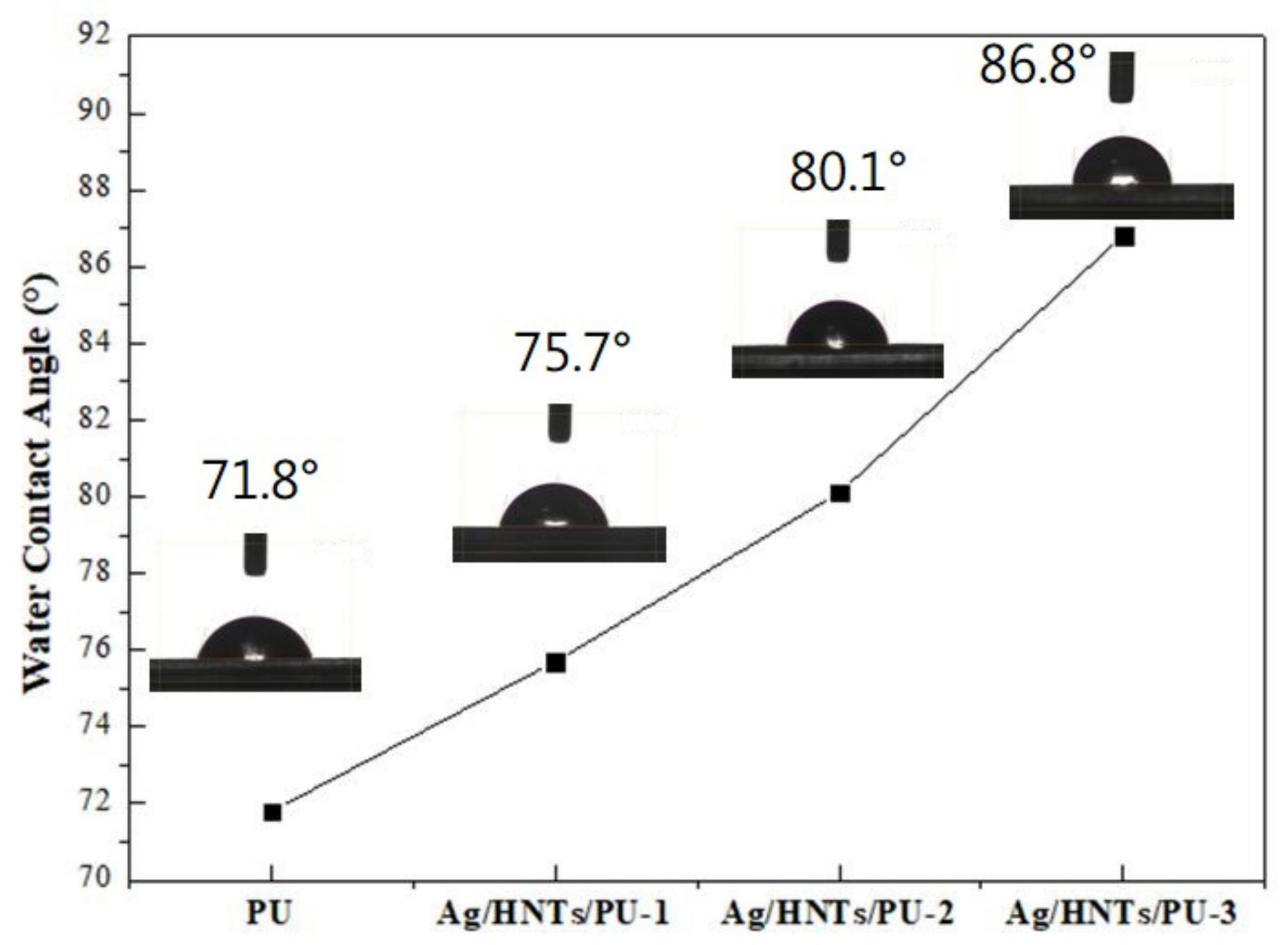
Water contact angle (WCA) trend chart of Ag/HNTs/PUs.

The 3D morphologies of the surfaces of Ag/HNTs/PUs are presented in Figure 10. The calculated average surface roughness of pure the PU, Ag/HNTs/PU-1, Ag/HNTs/PU-2, and Ag/HNTs/PU-3 were 0.37 nm, 2.79 nm, 3.16 nm, and 4.52 nm, respectively. The surface roughness of the PU thin films increased with the amount of Ag/HNTs. A possible reason is that increasing Ag/HNTs increased the amount of Ag/HNTs exposed on the surface of the thin films; this in turn resulted in the phenomenon increased average surface roughness. Figure 11 contains a water contact angle trend chart for the Ag/HNTs/PUs. The water contact angles of pure PU, Ag/HNTs/PU-1, Ag/HNTs/PU-2, and Ag/HNTs/PU-3 were 71.8°, 75.7°, 80.1°, and 86.8°, respectively. The Ag/HNTs thus increased the surface water contact angle of the PU thin films to as high as 86.8°. This indicated that the addition of Ag/HNTs help enhance the hydrophobic property of PU thin films, and this may be caused by the influence of surface roughness. This phenomenon was proven by AFM; the level of surface roughness increased with the addition of Ag/HNTs, and this in turn affected the surface water contact angle.

### 3.9. Antibacterial Evaluation of Ag/HNTs/PU Nanocomposites

**Figure 12:**
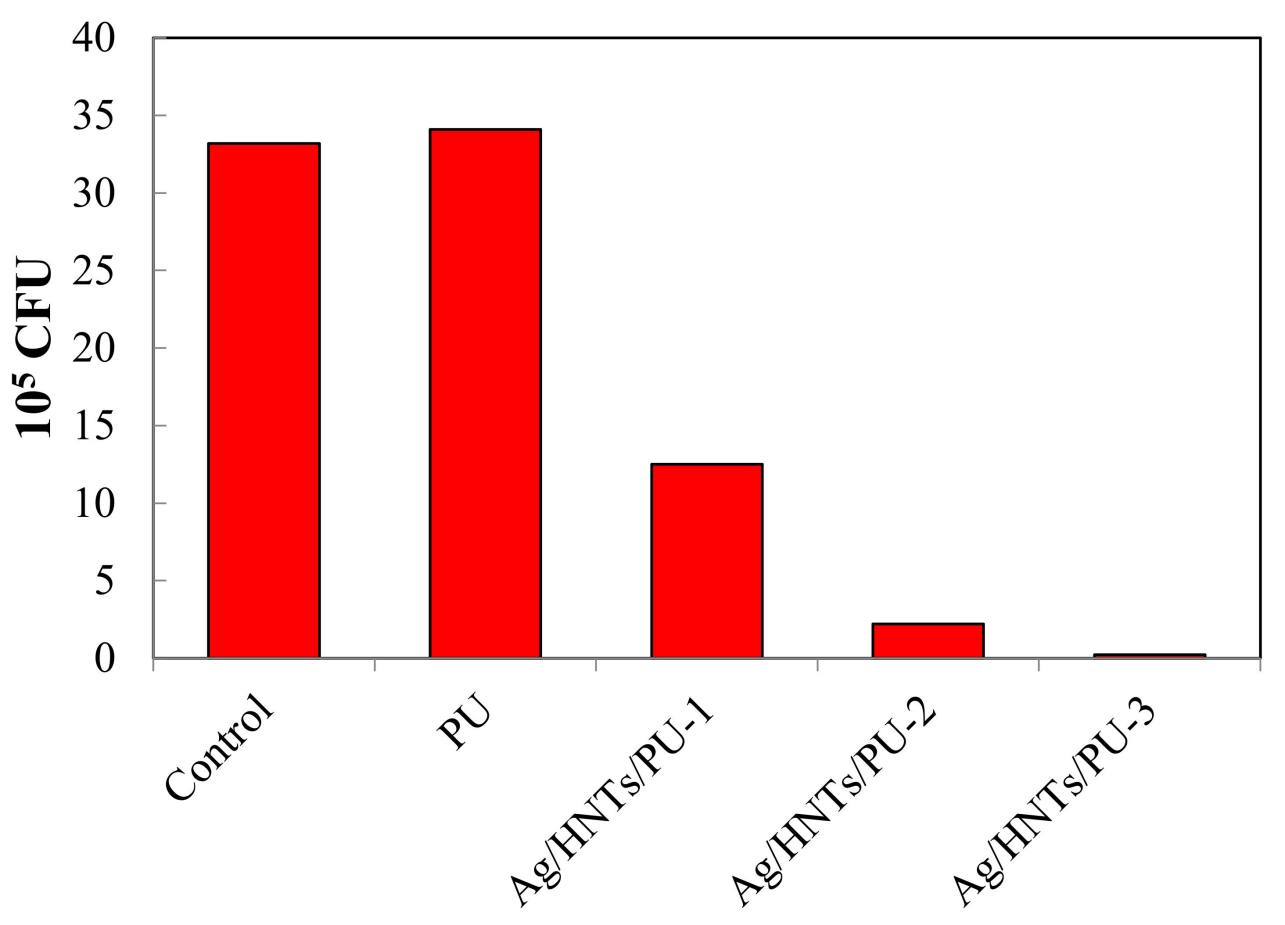
Bar chart illustrating the antibacterial effect of Ag/HNTs/PU on *E. coli*, based on the quantitative analysis conducted.

Figure 12 features a bar chart that illustrates the antibacterial effect of the Ag/HNTs/PU nanocomposites on *E. coli*. Quantitative analysis was conducted, and the relevant data are presented in Table 6. The test was to ASTM E2149-01 standard; 1 g samples of PU and Ag/HNTs/PUs were put in a 25 mL liquid petri dish containing *E. coli* bacteria liquid with a concentration of 1 × 10^5^ CFU/mL. They were placed in an incubator at 37 °C for 24 h. Then, 5 uL of bacterial solution for serial dilution was smeared on a plate, and the plate was placed in an incubator at 37 °C for 24 h, and finally the number of colonies was counted. The results indicated that pure PU does not possess any antibacterial property. However, antibacterial activity increased dramatically with the addition of Ag/HNTs. When Ag/HNTs content was 0.5 wt% and 1 wt%, 62.3% and 93.4% of *E. coli* were eliminated; when the content of Ag/HNTs was 2 wt%, 99.3% of *E. coli* were eliminated. Thus, the addition of Ag/HNTs endowed the PU with antibacterial effects; this is mainly because nanosilver itself possesses excellent antibacterial properties. The inorganic substance Ag/HNTs, formed through the coating of nanosilver on HNTs through a nanosilver coating technique, both helps in cost reduction and provides an excellent sterilization capacity. This result implies its potential in the field of biomedicine materials. To support these claims, we have summarized the relevant analytical parameters for antibacterial rate of *E. coli.* in Table 7, including the nanomaterial, substrate, addition method, antibacterial rate for various Ag-related nanomaterials.

## 4. Conclusions

In this study, the researchers prepared Ag/HNTs/PU nanocomposite materials using MDI, PTMG, 2,6-PDM, and silver-coated Ag/HNTs. FT-IR, TEM, XRD were used to determine whether the nanoparticles were coated successfully on the surface of halloysite such that Ag/HNTs could be produced and compounded into PU. The results of mechanical property analysis indicated that compared with pure PU, PU added with Ag/HNTs exhibited a 109.5% increase in tensile strength. TGA, DSC, and DMA analyses revealed that Ag/HNTs enhanced the thermal stability of PU; this was indicated by the increase of initial decomposition temperature from 291.0 °C to 330.1 °C and the increase of glass transition temperature from −51.5 °C to −47.4 °C. AFM analysis and water contact angles indicated that the hydrophobic property of Ag/HNTs/PU was increased (due to increased surface roughness). Furthermore, 99.3% of *E. coli* were eliminated when the Ag/HNTs additive was 2.0 wt%. This indicated halloysite containing Ag may cause Ag/HNTs/PU to have a high level of antibacterial activity, implying the potential of this type of material in the field of biomedicine.

## Figures and Tables

**Table 1:** Experimental formula of Ag/HNTs/PU.

Sample Name	MDI (mol)	PTMG (mol)	2,6-PDM (mol)	Ag/Halloysite (wt%)
PU	4	3	1	0
Ag/HNTs/PU-1	4	3	1	0.5
Ag/HNTs/PU-2	4	3	1	1
Ag/HNTs/PU-3	4	3	1	2

**Table 2:** The EDS results of Ag/HNTs and Ag/HNTs/PU.

Sample	Ag (wt%)
Ag/HNTs	13.96
Ag/HNTs/PU-1	1.02
Ag/HNTs/PU-2	1.95
Ag/HNTs/PU-3	3.96

**Table 3:** Thermal properties of the Ag/HNTs/PUs.

Sample	TGA		DSC
	T_onset_ (°C)	Residue at 700 °C	T_g_ (°C)
PU	291.0	0.2%	-51.5
Ag/HNTs/PU-1	323.7	4.1%	-50.7
Ag/HNTs/PU-2	329.0	5.4%	-48.9
Ag/HNTs/PU-3	330.1	6.1%	-47.4

**Table 4:** Dynamic mechanical analysis (DMA) results of the Ag/HNTs/PUs.

Sample	T_gd_ From E’’ (°C)	T_gd_ From Tanδ(°C)	Tan δ_max_
PU	-44.09	-35.68	0.5568
Ag/HNTs/PU-1	-42.27	-33.77	0.2854
Ag/HNTs/PU-2	-37.68	-32.73	0.2772
Ag/HNTs/PU-3	-33.72	-27.90	0.2530

**Table 5:** Tensile properties of the Ag/HNTs/PUs.

Sample	Tensile Strengths (MPa)	Strain at Break (%)	Young’s Modulus (MPa)
PU	10.5	1060	0.78
Ag/HNTs/PU-1	13.9	814	1.46
Ag/HNTs/PU-2	16.4	791	1.76
Ag/HNTs/PU-3	22.0	618	2.82

**Table 6:** Quantity of residual bacteria and antibacterial rate, as revealed by the quantitative analysis of Ag/HNTs/PU versus *E. coli.*

Sample	10^5^ CFU	Antibacterial Rate (%)
Control	33.2	-
PU	34.1	0
Ag/HNTs/PU-1	12.5	62.3
Ag/HNTs/PU-2	2.2	93.4
Ag/HNTs/PU-3	0.24	99.3

**Table 7:** The antibacterial rate of *E. coli*. in the literature and our work.

Nanomaterial	Substrate	Addition Method	Antibacterial Rate	Reference
Ag-HNT	WPU	Mix	94.69	[35]
Ag@SiO2	polyamide/polysulfone (PA/PSf)	Deposition	92.7 ± 1.8	[36]
Ag	PAN	Coating	99.7	[37]
Ag	PSf	In situ	97.6 ± 2.8	[38]
Ag	lysozyme/tannic acid	In situ	98.9	[39]
Ag/graphene oxide	melamine sponge	In situ	99.8	[40]
Ag-HNT	PU	Mix	99.3	our work
